# Inferior mesenteric vein thrombosis and COVID-19

**DOI:** 10.1590/0037-8682-0412-2020

**Published:** 2020-09-21

**Authors:** Aureo Carmo, Bruno da Silva Cunha

**Affiliations:** 1 Universidade Federal do Estado do Rio de Janeiro, Departamento de Terapia Intensiva, Rio de Janeiro, RJ, Brasil.; 2 Universidade Federal do Estado do Rio de Janeiro, Departamento de Cardiologia, Rio de Janeiro, RJ, Brasil.

A 33-year-old obese patient (body mass index=32.7), without other comorbidities, was admitted to our hospital with complaints of severe low back pain radiating to the hypogastric region. The pain had started about 8 hours before admission. Additionally, 11 days before admission, he had experienced dry cough, a fever of 38.2°C, and fatigue and was diagnosed with SARS-CoV-2 infection by nasopharyngeal swab testing.

**FIGURE 1:**
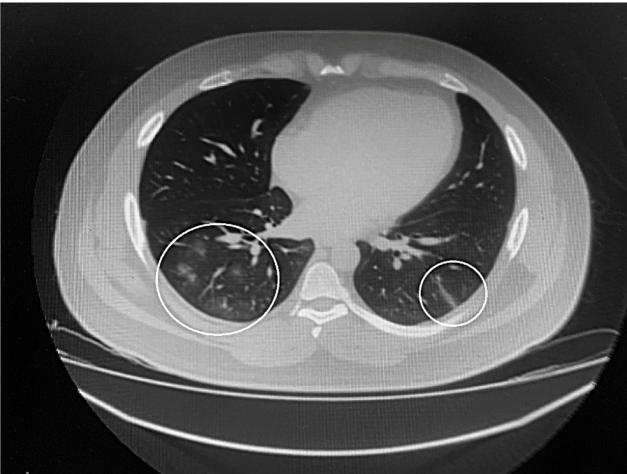
Peripheral ground-glass pattern affecting both lower lobes, suggestive of viral pneumonia.

**FIGURE 2:**
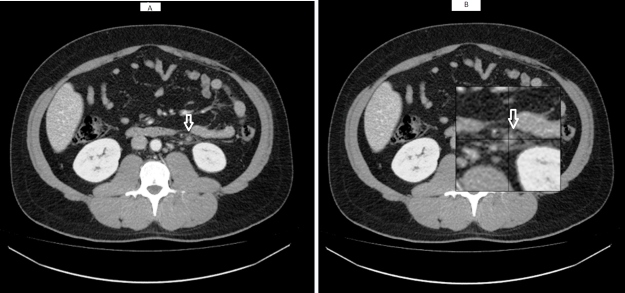
(**A**) Enlarged inferior mesenteric vein that is not completely filled by contrast associated with infiltration of the adjacent adipose planes denoting mesenteric thrombosis; (**B**) magnified image.

He presented without abdominal distension or signs of peritonitis. His vital signs were normal. Blood tests demonstrated elevations in D-dimer (879 ng/mL), ferritin (1570 ng/mL), and C-reactive protein (58.2 mg/dL). Chest computed tomography (CT) showed infiltration in a peripheral ground-glass pattern affecting both lower lobes, suggestive of viral pneumonia (Figure 1). Abdominal CT scan showed an enlarged inferior mesenteric vein not completely filled by contrast associated with infiltration of the adjacent adipose planes, thus denoting mesenteric thrombosis (Figure 2).

The patient was administered saline and analgesics. Complete anticoagulation was performed with enoxaparin. About 24 hours after admission, complete remission of pain was observed, and an oral diet was restarted. After five days of parenteral anticoagulant treatment, oral warfarin was started. Two days later, with the International Normalized Ratio at 2.3, the patient was discharged from hospital.

Mesenteric venous thrombosis is a rare condition, estimated to occur in 0.002%-0.06% of hospital admissions^1^ and unlike mesenteric arterial thrombosis, is associated with prothrombotic and primary states of hypercoagulability. Thrombosis in atypical sites associated with COVID-19 has also been described, and the mechanisms suggested are direct damage, hemodynamic impairment, and thrombosis^2^.
